# Eccrine Sweat Gland and Its Regeneration: Current Status and Future Directions

**DOI:** 10.3389/fcell.2021.667765

**Published:** 2021-07-28

**Authors:** Yao Lin, Liyun Chen, Mingjun Zhang, Sitian Xie, Lijie Du, Xiang Zhang, Haihong Li

**Affiliations:** ^1^Department of Plastic Surgery and Burn Center, Second Affiliated Hospital of Shantou University Medical College, Shantou, China; ^2^Department of Wound Repair and Dermatologic Surgery, Taihe Hospital, Hubei University of Medicine, Shiyan, China

**Keywords:** eccrine sweat gland, regeneration, stem cells, scaffolds, signaling pathways, methods

## Abstract

Eccrine sweat glands (ESGs) play an important role in temperature regulation by secreting sweat. Insufficiency or dysfunction of ESGs in a hot environment or during exercise can lead to hyperthermia, heat exhaustion, heatstroke, and even death, but the ability of ESGs to repair and regenerate themselves is very weak and limited. Repairing the damaged ESGs and regenerating the lost or dysfunctional ESGs poses a challenge for dermatologists and bum surgeons. To promote and accelerate research on the repair and regeneration of ESGs, we summarized the development, structure and function of ESGs, and current strategies to repair and regenerate ESGs based on stem cells, scaffolds, and possible signaling pathways involved.

## Introduction

As warm-blooded animals, humans regulate body temperature through various regulatory mechanisms. Among them, ESGs play an important role in cooling down body temperature by secreting primarily water that contains electrolytes (Saga, 2002). Human skin has two major types of sweat glands: eccrine and apocrine. The apocrine sweat glands are appendage of the hair follicle and release a cloudy, viscous fluid through the follicle orifice, which exclusively present in highly localized hairy axillary regions, and they are non-thermoregulatory (Sato et al., 1989). Some patients lack ESGs due to severe burns or genetic factors, while some patients suffered from congenital or acquired factors resulting in ESG dysfunction. If the human body has no way to sweat, it means that any hot weather or acute activity can cause them to get heatstroke or even die. Therefore, we focus on the wound repair and regeneration of ESGs in this review.

First, it is necessary to understand the normal structure and functions of ESGs. On the surface of the body, ESGs are small but very numerous (Sato et al., 1989), which directly open to the skin surface. During exercise, fever or hot environments, humans are able to dissipate heat through sweat to maintain body temperature within the optimal range (Shibasaki et al., 2006). In contrast, for most domestic mammals, most of their body surface lack ESGs. Mouse is the common model for ESG study because of the similarity of human ESG structure and function, which has ESGs solely present in the pads of their paws (Lu et al., 2012).

**FIGURE 1:**
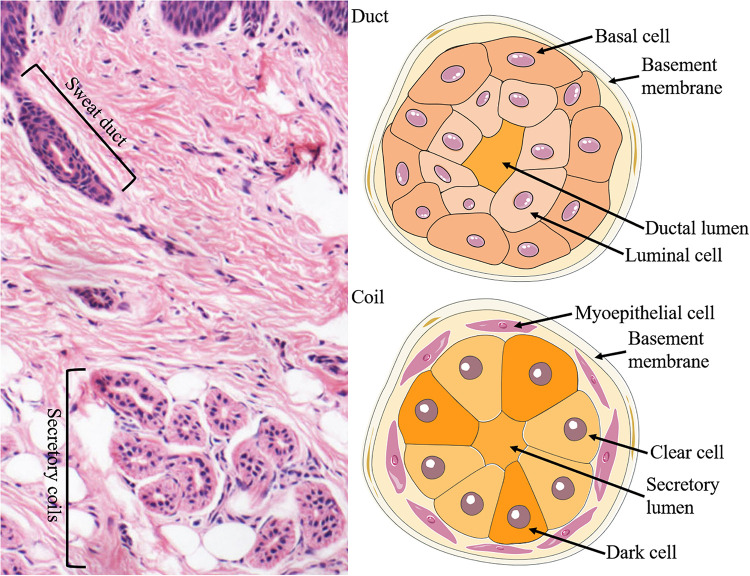
Structure and cellular constituents of ESGs. The ESG is comprised of a relatively straight duct led to the skin surface and a secretory coil deep in the dermis (left panel). The duct is formed of two layers of cells: the basal (outer) and luminal (inner) cells, where ions are partially reabsorbed (right upper panel). There are three types of cells in the secretory coil: clear cells, dark cells, and myoepithelial cells (right lower panel).

The ESGs are small tubular structures situated in epidermis and dermis. They comprise a relatively straight duct led to the skin surface and a secretory coil deep in the dermis. The duct of the ESG is a straight channel, and the secretory portion of the ESG is a distinctive, coiled tubular structure (Figure 1).

There are three types of cells in the secretory coil: clear cells, dark cells, and myoepithelial cells. Myoepithelial cells provide power support for sweat secretion and support the glands mechanically (Sato, 1977; Sato et al., 1989). The secretory cells can be classified into clear cells and dark cells based on their affinities to basic dyes and granule contents (Montagna et al., 1953; Munger, 1961). The clear cells are without secretory granules but have many mitochondria and membrane villi, which contribute to generate water, electrolytes, and inorganic substances in the sweat. By contrast, the dark cells contain many Schiff-reactive granules, which are mainly in charge of generating macromolecules such as glycoproteins (LobitzJr., and Dobson, 1961; Munger, 1961; Yanagawa et al., 1986). Furthermore, sweat also contains various proteolytic enzymes (Horie et al., 1986), IgA (Okada et al., 1988), active interleukin-1 (Sato and Sato, 1994) and several antimicrobial peptides (Schittek et al., 2001; Niyonsaba et al., 2009), which likely to be conducive to the barrier function of the skin.

The development of electron microscopy (EM) and the ultrastructure that it revealed accelerated the studies of ESGs. Ultrastructural observations on the development of ESG in human embryos have been reported since the 1960s (Hashimoto et al., 1965). From the perspective of embryonic development, at 3 about months, the epidermal ridges on the palms begin to form epithelial cell cords, which are the starting point for the development of ESGs, and at 5 about months, ESGs in other parts of the body begin to develop (Sato et al., 1989). By the eighth month of the fetus, ESGs are morphologically mature (Sato et al., 1989). In mice, ESG germs were spotted at E17.5 and the coiling of secretory portions was at P1, and ESG formation was in essence completed by P5 (Kunisada et al., 2009; Figure 1). In rats, ESG germs were first detected at E19.5, straight ducts first appeared at E21.5, and secretory coils began to form at P1 (Li et al., 2017). During the ESG morphogenesis, the progenitor properties change from multipotency to unipotency, and ultimately, they form four unipotent adult stem cell populations: basal duct, suprabasal duct, myoepithelial, and glandular luminal stem cells (Lu et al., 2012). Proliferation is almost undetectable in the mature glands and remain active only in the basal cells of the sweat duct and the epidermis of the paw skin (Lu et al., 2012).

## Feasibility of Regeneration of ESG

**FIGURE 2:**
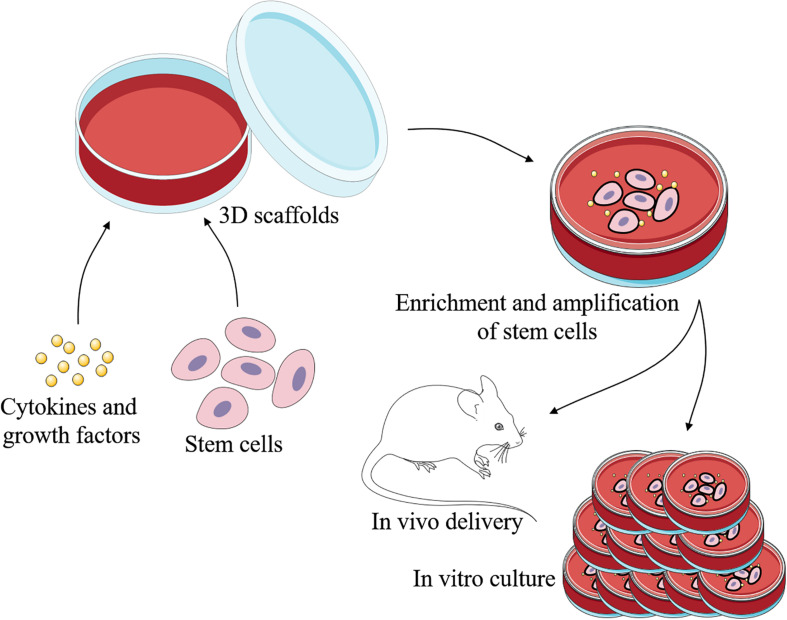
Schematic representation of regeneration of ESGs. With the 3D scaffolds, specific cells can be induced by specific cytokines and growth factors to differeniate into sweat gland-like cells. There are three main types of cells that may be used to repair and regenerate ESGs: ESG-derived stem cells, non-sweat gland-derived stem cells, and induced pluripotent stem cells. This process can take place *in vivo* or *in vitro*.

**FIGURE 3:**
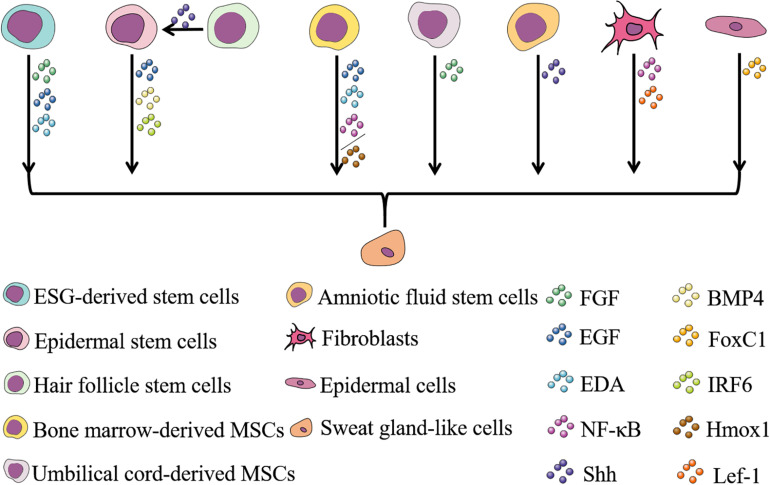
Cells and factors involved in ESG regeneration and their relationships. Stem cells are often used in the study of wound repair and regeneration of various tissues due to their ability to self-renew and differentiate into multiple lineages. To date, eight types of cells have been reported to have the potential to regenerate ESGs (Shikiji et al., 2003; Li et al., 2006, 2015b; Nolte et al., 2008; Sheng et al., 2009; Vierbuchen et al., 2010; Lu et al., 2012; Xu et al., 2012, 2016; Wang et al., 2013, 2019; Hassan et al., 2014; Tao et al., 2014; Liang et al., 2016; Kolakshyapati et al., 2017; Sun et al., 2018; Yao et al., 2018, 2019, 2020; Chen et al., 2019; Hu et al., 2019). Cytokines and growth factors play a role in inducing cells directed to differentiate into sweat gland-like cells during the process of ESG regeneration, and the discovery of these factors involves research on the determination and development of ESGs (Shikiji et al., 2003; Cai et al., 2011; Zhao et al., 2015; Liang et al., 2016; Xu et al., 2016; Kolakshyapati et al., 2017; Sun et al., 2018; Yao et al., 2018, 2019, 2020; Chen et al., 2019; Hu et al., 2019; Wang et al., 2019). Selecting appropriate cells and appropriate factors to induce the differentiation of sweat gland-like cells makes the regeneration of ESGs possible.

Engineered skin is certainly developing rapidly today, while it still lacks skin appendages. As skin appendages, ESGs play important roles in the temperature regulation and maintenance of homeostasis (Huang et al., 2010). So far, patients with irreversible loss of functional ESGs still cannot receive effective treatment. Current strategies for repair and regeneration of ESGs are mainly based on stem cells, scaffolds, bioactive cytokine and growth factors, and involved signaling pathways (Figures 2, 3).

### ESG Regeneration by Stem Cells

Adult tissue-specific stem cells are distributed in various tissues and organs. In the skin, stem cells have long been found in the epidermis and hair follicles, but it was not known until recently that ESGs are also rich in stem cells (Lu et al., 2012). As judged from immunohistochemical staining of nucleotide analog incorporation and cell proliferation markers, proliferation occurs rarely in the secretory coil cells, but frequently in the basal cells of sweat ducts during homeostasis of adult ESGs (Morimoto and Saga, 1995; Li et al., 2008, 2016b; Chen et al., 2014). With the use of lineage tracing and pulse-chase studies, ESG stem cells have been identified from both developing and mature mouse ESGs by Lu et al. (2012). The multipotent K14^+^ bud progenitors in the basal layer of embryonic ectoderm is the starting point of ESG formation, which then develops into transient multipotent K14^+^ basal progenitors and K18^+^/lowK14 suprabasal progenitors (Lu et al., 2012). Finally, in mature ESGs, the progenitor properties change from multipotency to unipotency in the form of four unipotent adult stem cell populations: basal duct, suprabasal duct, myoepithelial, and glandular luminal stem cells (Lu et al., 2012).

Basal cells in paw epidermis and sweat ducts proliferate can renew and replenish cells of scuffed suprabasal epidermis and intraepidermal duct during homeostasis (Lu et al., 2012; Chen et al., 2014; Li et al., 2016b). When epidermis is severely damaged or excised, neighboring basal cells of epidermis and sweat duct, not including secretory coil cells, rapidly proliferate to repair the injured area (Lu et al., 2012; Chen et al., 2014; Li et al., 2016b). The basal and suprabasal duct stem cells also contribute to repair the skin epidermis and epidermal sweat ducts wound (Lu et al., 2012; Chen et al., 2014; Li et al., 2016b).

There have been many studies that have shown the quiescent nature of both luminal and myoepithelial cells of the secretory coil in adult ESGs (Li et al., 2008, 2016b; Lu et al., 2012). Only when localized injury occurs, do myoepithelial and glandular luminal progenitors replenish their own descendants, and the remarkable thing is that they act as unipotent progenitors during repair (Lu et al., 2012). Luminal cells can proliferate to repair neighboring injured luminal cells, and myoepithelial cells can proliferate to repair neighboring injured myoepithelial cells (Lu et al., 2012). Many studies have shown that the myoepithelial cells of adult ESGs are quiescent (Li et al., 2008, 2016b; Lu et al., 2012).

There are also studies showing that ESG secretory cells not only participate in their own repair, but also participate in the repair of the epidermis, and their regeneration and repair ability is stronger than that of sweat duct luminal cells (Rittie et al., 2013; Pontiggia et al., 2014; Diao et al., 2019). As for myoepithelial cells, it is not clear whether they are involved in epidermal repair under physiological conditions. However, studies have shown that engrafting purified myoepithelial cells to back skin can generate epidermis (Lu et al., 2012). Investigators also isolated cells with typical characteristics of mesenchymal stem cells, from myoepithelial cells of secretory coils in adult human ESGs, which may contribute to the study of wound repair and ESG regeneration (Kurata et al., 2014; Ma Y. et al., 2018).

As is mentioned above, the stem cell populations in mature ESGs are unipotent. However, some unipotent stem cells tend to regain multipotency when leaving the original environment. Based on cell-surface markers, Lu et al. (2012) exploited fluorescent activating cell sorting (FACS), purified different cell populations from mouse secretory coils and sweat ducts, and studied their individual regenerative capacities in engraftment experiments. Grafting the myoepithelial or basal duct stem cells, but not luminal or suprabasal duct stem cells, into cleared mammary fat pads or shoulder fat pads can regenerate *de novo* ESGs (Lu et al., 2012). Notably, there have been many studies that have shown the quiescent nature of myoepithelial cells in adult ESGs (Li et al., 2008, 2016b; Lu et al., 2012; Leung et al., 2013). Based on these findings, it is interesting that adult progenitors show single-function nature in their native environmemt. Therefore, further experiments will be needed to analyze the molecular causes.

In a previous *in vitro* study, Li et al. (2013) demonstrated that human ESG cells cultured in Matrigel not only build three-dimensional (3D) tubular-like structures with lumens, but also express α-SMA, epithelial membrane antigen (EMA), CK7, and CK19, and then, they did *in vivo* experiment on this basis, Matrigel-embedded ESG cells were subcutaneously implanted into nude mice (Li et al., 2015a). Compared with ESGs formed *in vitro*, ESGs formed in nude mice were more similar to natural ones (Li et al., 2015a). Reconstituted 3D ESGs recapitulated the polarization at the appropriate time points during spheroid differentiation, and secreted fluid similar to native human ESGs (Li et al., 2016a). In addition to the above, the authors also demonstrated that the 3D-reconstituted ESGs were nourished by blood vessels and mediated by both cholinergic and adrenergic innervation (Zhang et al., 2018). Thus, the 3D-reconstituted ESGs have the completeness of structural components, the prerequisite for full functionality, from which the authors inferred that the 3D-reconstituted ESGs may function as the native ones do. However, the secretory function of the 3D-reconstituted ESGs remains to be fully established. All in all, it is an intriguing development in the process of questing treatments burn patients.

The difficulty of using isolated ESG cells to reconstruct sweat gland-like (SGL) structures is that ESG cells are dispersive in the dermis and difficult to gather. Further, with extensive severe burns, the ESGs of patients are destroyed and autologous mature ESG cells and ESG stem cells are insufficient. The optimized cell culture of Diao et al. (2019) can provid the appropriate cells in sufficient quantity for mouse ESGs and skin regeneration, and offers a new strategy for regenerating SGL structures.

In skin tissues, epidermal stem cells (EpiSCs), as the specific stem cell type, can regenerate skin tissue, repair wound and re-modeling (Boehnke et al., 2012). During embryonic development, both ESGs and hair follicles (HFs) originate from EpiSCs, so EpiSCs are the common progenitor cells of both ESGs and HFs. Research has shown that young human keratinocytes, including EpiSCs, can invade collagen gels and differentiate into/toward ESG duct-like structures *in vitro* with fibroblasts, epidermal growth factor (EGF) and fetal bovine serum (FBS) (Shikiji et al., 2003). EGF, interferon regulatory factor 6 (IRF6) and bone morphogenetic protein 4 (BMP4) have also been shown to play a role in inducing EpiSCs to transform into ESG cells (Shikiji et al., 2003; Yao et al., 2018; Hu et al., 2019). Therefore, EpiSCs can be induced directly and differentiate into ESG cells, and is one of the most common means of ESG regeneration. However, in the adult body, the number of EpiSCs is limited, for merely 1–10 percent of basal stem cells (Cotsarelis et al., 1999). As a result, producing a large number of SGL cells (SGLCs) by epidermal cell reprogramming may be another method for ESG regeneration. Yao et al. (2019) showed that overexpressing the transcription factor FoxC1 can directly reprogram epidermal cells to induce functional SGLCs. Since the epidermis of patients with extensive severe traumatic burns is damaged and autologous mature epidermal cells and EpiSCs is scarce, this method of regeneration is more suitable for anhidrotic/hypohidrotic ectodermal dysplasia patients (Yao et al., 2019).

Bone marrow-derived MSCs (BM-MSCs) are characterized by lower immunogenicity and rarely destroyed in the event of skin damage, so they have great potential for development (Zhang et al., 2015). Although the mechanism of using BM-MSCs to regenerate ESGs remains unclear, multiple cytokines appear to play an important role in ESG regeneration and development. Li et al. (2006) directly co-culture BM-MSCs with heat-shocked ESG cells and found that it can differentiate BM-MSCs into SGLCs. Then, transplanting SGLCs into the wounds of nude mice showed a significantly promotion of damaged ESG repair and regeneration (Sheng et al., 2009). Li et al. (2015b) have also demonstrated that 3D co-culture of BM-MSCs and ESG cells in Matrigel can help the transdifferentiation of BM-MSCs into ESG cells, with the transdifferentiated BM-MSCs potentially able to function as ESG cells. There are other ways to directly induce BM-MSCs to differentiate into SGLCs, and involves various cytokines and scaffolds, which will be described in the following chapters. Even though there is a distinct advantage using BM-MSCs for ESG regeneration, the number of BM-MSCs is limited and it is difficult to maintain pluripotency after extensive passage (Zhang et al., 2015). Recently, investigators have reported that severely burned skin contains viable, undamaged cells that show characteristics of human MSCs, and can be used to promote wound healing without adverse side effects (Amini-Nik et al., 2018). These findings provide an ideal source of MSCs for treatment of severely burned patients.

### 3D Reconstitution Model of ESG *in vitro/vivo*

The extracellular matrix (ECM), often used to refer to all the substances surrounding cells in a multicellular organism except for circulating fluids, is a 3D structural scaffold made of non-cellular, fibrous, and non-fibrin proteins that exists in all tissues and is a major component of the cellular microenvironment (Theocharis et al., 2016). The ECM does more than provide physical support for organizational integrity and resilience: it is a dynamic structure that is constantly reshaped to control organizational homeostasis and organ development, as well as tissue repair and regeneration (Bonnans et al., 2014). A highly dynamic 3D ECM provides environmental signals that influence basic cell behaviors, such as cell proliferation, adhesion, migration and differentiation, impact cell mechanics, and regulate the fate of stem cells (Watt and Huck, 2013). Therefore, the ECM plays essential roles not only in embryonic development and homeostasis, but also in tissue engineering and regenerative medicine (Blankenship, 1990; Watt and Huck, 2013; Bonnans et al., 2014). 3D scaffolds are manufactured by removing cellular content from source tissues while retaining the original structural and functional molecular units of the ECM, and it has been widely applied to the field of tissue engineering and regenerative medicine (Costa et al., 2017).

So far, the studies on isolated sweat gland stem cells/progenitor cells cultured in traditional monolayers have always rapidly differentiated into keratinocytes and lost their specific phenotypic characteristics (Rittie et al., 2013; Pontiggia et al., 2014). Compared with the traditional 2D culture models, 3D culture models recapitulate the function and physiological architecture of the body (Kleinman and Martin, 2005; Kozowski et al., 2011). Under 2D culture conditions, cells undergo proliferation but have difficulty in inducing directional differentiation, but under 3D culture conditions, they could be induced directional differentiation (Petrakova et al., 2012; Li et al., 2015b). Therefore, culturing cells under 3D conditions is a useful model for studying cell proliferation and differentiation. To date, researchers have developed several kinds of 3D organoid culture matrices for ESG regeneration, aiming to achieve the enrichment and amplification of cells while maintaining the specific characteristics of ESG cells.

The Matrigel basement membrane matrix (abbreviated as Matrigel) is a dissolved basement membrane preparation that contains fetal collagens, laminin, entactin, heparan sulfate proteoglycans, and several matrix-bound growth factors, which help cell growth as organoids (Kleinman and Martin, 2005; Li et al., 2015a). Using 3D culture method to culture cells in a gel basement membrane matrix, many cells will differentiate into tissue-specific structures, and vascular endothelial cells are one of the earliest cell types showing morphological differentiation (Kleinman and Martin, 2005; Arnaoutova et al., 2009). The differentiation of endothelial cells in Matrigel mimics the process of angiogenesis *in vivo*, which indicates that Matrigel can be used to obtain a large amount of information about angiogenesis regulators, genes that play an important role in angiogenesis in endothelial cells, and the characterization/identification of endothelial progenitor cells (Auerbach et al., 2003). Besides this, Matrigel has been widely used to study tumor cell invasion, and an altered ECM has been shown to promote tumorigenesis (Bissell and Labarge, 2005). Salivary gland cell lines cultured on Matrigel are widely used to study cell differentiation, such as glandular-like morphogenesis, acinus formation and branching morphogenesis (Barka et al., 2005). Maria et al. (2011) obtained cells from parotid and submandibular glands, expanded *in vitro*, and then cultured on Matrigel. On Matrigel-coated substrates, cells formed 3D acinar-like units, adopting a large number of secreted granular acinar phenotypes, expressing α-amylase and the water channel protein, aquaporin-5. Experiments by Kozowski et al. (2011) show that the bovine mammary epithelial cell line BME-UV1 cultured on Matrigel could form 3D acinar structures with a hollow lumen in the center, which is similar to the mammary gland alveoli in a functionally active mammary gland. To study ESG progenitor/stem cells, Lu et al. (2012) suspended four sorted ESG cells in Matrigel and injected them individually into cleared mammary or shoulder fat pads from female Nu/Nu mice. In rare cases, purified adult ductal basal cells produce glands and ducts, while purified myoepithelial cells continue to form ESGs, and luminal or suprabasal duct cells did not show this diverse behavior (Lu et al., 2012). Subsequently, Matrigel was applied to the regeneration of ESGs. Li et al. (2013, 2015a) inoculated ESG cells into the tissue structure formed by a Matrigel basement membrane matrix *in vitro* or in nude mice to simulate the growth microenvironment of natural ESGs, and successfully reconstructed SGL structures using the isolated ESG cells. These studies indicate that the interactions between Matrigel and ESG cells play important roles in the 3D reconstruction of SGL structures. On this basis, Diao et al. (2019) added some growth factors and small molecules, such as EGF, bFGF, and EDA, in order to increase the differentiation efficiency. Although there are some differences between the reconstructed SGL structures and the original ESGs, these studies demonstrated that Matrigel can induce ESG cells to reconstitute SGL structures. Maybe subsequent work could implant Matrigel-embedded ESG cells subcutaneously into burn victims to reconstitute ESGs. However, in practice, the implanted ESG cells do not reconstruct ESGs with complete structure and function as we had hoped. Therefore, in the following scientific research work, there are still many problems for us to explore and solve.

Three-dimensional bioprinting has become a promising technology for manufacturing complex tissue structures with tailor-made biological components and mechanical properties (Murphy and Atala, 2014). By using this revolutionary technology, bio-inks, including growth factors, cells, and hydrogels, can be precisely positioned to create 3D *in vitro* culture environments (Ma X. et al., 2018). Pati et al. (2014) decellularized adipose, cartilage and heart tissue to make bioink, and adopted a 3D bioprinting technique to construct a 3D structure *in vitro*, successfully inducing adipose-derived MSCs to express specific markers of cardiomyocytes and chondrocytes. By building 3D printing scaffolds that continuously release a variety of growth factors, Lee et al. (2014) successfully treated sheep with damaged menisci by inducing endogenous MSCs to differentiate into menisci *in vivo*. The findings strongly suggest that 3D bioprinting has great potential in simulating the microenvironment to induce stem cell differentiation and promote tissue regeneration. Through 3D bioprinting, Fu’s research team successfully induced EpiSCs to differentiate into ESG cells using gelatin-alginate hydrogels and mouse ESG-ECM protein components (Huang et al., 2016; Liu et al., 2016; Li et al., 2018). They subsequently adopted 3D bioprinting to mimic the regenerative microenvironment to direct of MPCs or MSCs to specifically differentiate into ESGs, and ultimately guide the formation and function of glandular tissue (Wang et al., 2019; Yao et al., 2020). Alginate/gelatin hydrogel can serve as bio-ink due to its good cell compatibility, printability, and stable structure during long-term culture (Huang et al., 2009). Wang et al. (2019) used gelatin-alginate hydrogels to combine with ESG-ECM protein to form a characteristic bio-ink, which made it possible to induce the transformation of mammary progenitor cells to ESG cells (Yao et al., 2020). Although its mechanism still needs further exploration, it may be used as an effective tool to induce ideal cells or tissues *in vitro* through an engineered microenvironment in the future.

Gelatin is not only an irreversible form of denatured collagen, it has the ability to form a scaffold suitable for dermal regeneration without adding any other polymers, but also has the ability to control the release of growth factors for a long time (Shevchenko et al., 2014). Therefore, Huang et al. (2009, 2010) developed gelatin microspheres containing EGF as multifunctional vehicles on which ESG cells could be cultured, and delivered these ESG cell-microsphere complexes into an engineered skin for wound repair. Later, they delivered BM-MSCs by an EGF microsphere-based engineered skin model to repair ESGs and improve cutaneous wound healing (Huang et al., 2012). Analogously, Kolakshyapati et al. (2017) combined the collagen-chitosan porous scaffold with Lipofectamine 2000/pDNA-EGF complexes to yield a gene-activated scaffold (GAS) on which BM-MSCs are cultured. Such GAS/BM-MSCs could accelerate the wound healing and induce full-thickness skin regeneration with SGL structure *in situ* (Kolakshyapati et al., 2017). These engineered skin constructs are promising tools for ESG regeneration in skin repair and are a valuable engineering strategy for constructing engineered skin models containing appendages.

## Mechanism of ESG Development and Regeneration

**FIGURE 4:**
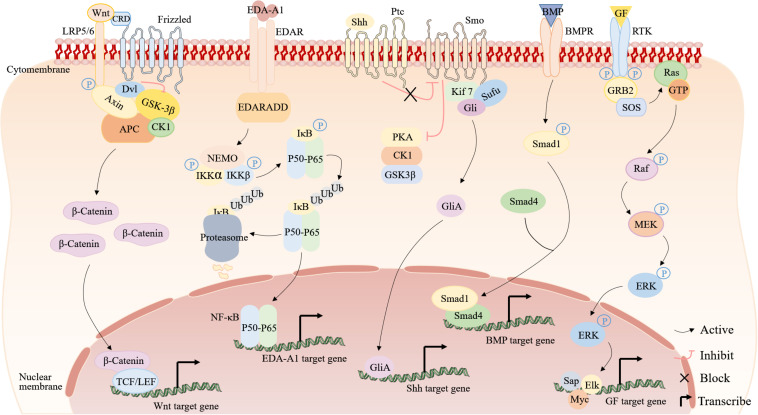
The signaling pathways involved in ESG development and regeneration. From left to right are the Wnt, EDA-A1, Shh, BMP, and ERK signaling pathways. Wnt signaling pathway (Nusse and Clevers, 2017; Routledge and Scholpp, 2019): in the absence of Wnt signals, GSK-3β/Axin/APC/β-catenin/CK1 is a destruction complex. GSK-3β phosphorylates β-catenin, thereby inhibiting its activity and then β-catenin is degraded by ubiquitination. When Wnt proteins bind to a receptor complex, it induces the association of Axin and Dvl with LRP5/6 and Frizzled. Dvl inhibits GSK-3β and the destruction complex falls apart, and thus prevents the degradation of β-catenin, permitting β-catenin accumulation, leading to binding TCF/LEF in the nucleus to upregulate target genes. EDA signaling pathway (Oeckinghaus et al., 2011; Sisto et al., 2016): in the absence of EDA-A1 signals, NF-κB dimers (P50–P65) are bound to inhibitory IκB proteins, which sequester inactive NF-κB complexes in the cytoplasm. When EDA-A1 binds to EDAR, stimulation of EDAR leads to the binding of EDARADD, for IKK activation. Shh signaling pathway (Fattahi et al., 2018; Xin et al., 2018): in the absence of Shh signals, Ptc inhibits the activity of Smo by affecting its localization to the cell surface, and protein kinases (PKA, CK1, and GSK3β) constitutively phosphorylate Gli proteins to inhibit the Gli. The secreted active Shh ligand binds to Ptc and relieves the repressive effect of the Ptc on Smo, activating the Smo, which then translocates to the cell membrane to inhibit PKA, CK1, and GSK3, providing an assembly platform for the recruitment of Kif7, Sufu and Gli, thus activating the Gli. Subsequently, the activated Gli forms (GliAs) translocate into the nucleus and activate Shh target genes. BMP signaling pathway (Gonzalez and Medici, 2014): when BMPs bind to BMPRs, intracellular Smad1 becomes phosphorylated. The phosphorylated Smad1 binds to Smad4 and then translocates into the nucleus and activates BMP target genes. ERK signaling pathway (Calvo et al., 2010; Gallo et al., 2019): Phosphorylated RTK binds to GRB2, and GRB2 binds to SOS, which stimulates RAS. RAS initiates activation of the MEK-ERK cascade by converting a molecule from GDP to GTP.

Up to now, studies have revealed involvement of Wnt, EDA, Shh, BMP, and ERK signaling pathways in ESG determination and development (Figure 4). These findings lead to a series of explorations into the regeneration of ESG.

### Wnt/β-Catenin Signaling Pathway

Wnt/β-catenin signaling pathway is a relatively conservative cell-cell communication system in evolution, which is very important for embryogenesis, stem cell renewal, cell proliferation and cell differentiation (Steinhart and Angers, 2018). When cytokines activate the Wnt signaling pathway, β-catenin accumulates and enters the nucleus, associates with DNA binding factors of the TCF/LEF family, and activates the expression of target genes (Xu et al., 2017). The Wnt/β-catenin signaling pathway is active in the appendages of embryonic ectoderm and is necessary for their formation. Whether the Wnt signaling is upstream or downstream of the EDA signaling is controversial in the basal formation process of the ectodermal appendage, but now, there is mounting evidence that Wnt signaling is an upstream regulator of EDA signaling (Cui et al., 2014). As ESG germs start to form, Wnt activity declines quickly in the dermis and rises strongly in the basal layer of epidermis, and then stays active at the tip of the growing ducts until it disappears when the sweat ducts starts to coil (Cui et al., 2014). According to reports, Wnt10a mutations account for 16% of human hyperhidrosis ectodermal dysplasia (HED) patients (Cluzeau et al., 2011). After further study, researchers have found that Wnt10a/β-catenin signaling is necessary for ESG germ development and postnatal ESG duct development (Xu et al., 2017). It will be interesting in the future to apply Wnt10a to ESG regeneration.

### EDA/EDAR/NF-κB Signaling Pathway

Hypohidrotic ectodermal dysplasia is a well-characterized human disease characterized by absent or malformed HFs, teeth, and ESGs (Cui and Schlessinger, 2006; Mikkola, 2009). Much of the information known about ESG determination and development related to signaling pathways originated from research on HED patients. As a member of the TNF family of signaling molecules, ectodysplasin-A (EDA) exists as two highly homologous isoforms, EDA1 and EDA2, and the EDA-A1 gene, specific for the type I transmembrane protein EDA receptor (EDAR), is one of the genes that regulates the determination and development of ESGs (Srivastava et al., 2001). The main axis of the pathway comprises EDA (encoded in mice by tabby), EDAR (encoded by downless), and EDAR-associated death domain (EDARADD, encoded by crinkled) (Srivastava et al., 1997; Monreal et al., 1999). Any mutation in the components of these pathways will cause HED, which is phenocopied in mice (Headon et al., 2001; Cui and Schlessinger, 2006). In addition, mice deficient for nuclear factor-κB (NF-κB) activity also showed a phenotype identical to HED, leading researchers to realize that EDA/EDAR sends signals through the NF-κB pathway during skin appendage development (Doffinger et al., 2001; Kumar et al., 2001; Schmidt-Ullrich et al., 2001). Studies have found that EDA mainly regulates ESG maturation through activating NF-κB after binding to EDAR in the early stages of embryonic development (Doffinger et al., 2001; Kumar et al., 2001).

The almost complete restoration of ectodermal appendages (including ESG) is caused by the transgenic expression of the mouse EDA-A1 isoform in Tabby (EDA-less) (Srivastava et al., 2001), but wild-type mice overexpressing EDA-A1 showed larger ESGs with greater activity (Mustonen et al., 2003). Furthermore, Gaide et al., found that treating pregnant Tabby mice with EDA-A1 recombinant protein can permanently rescue the tabby defect in the offspring (Gaide and Schneider, 2003). Thus, researchers have hypothesized that activation of the EDA gene could induce the regeneration of ESGs. In support of this, the reprogramming of BM-MSCs to SGLCs was successfully induced by the high expression of EDA gene in BM-MSC (Cai et al., 2011). In addition, the findings of Sun et al. (2018) demonstrate that induction of EDA gene overexpression via transfection with an RNA-guided dCas9-effector could promote the transformation of BM-MSCs into SGLCs. These results indicate that the potential of EDA-modified MSCs for the repair and regeneration of ESGs.

As downstream effectors of EDA and EDAR signaling, IKK pathway activates the NF-κB transcription factors for development of skin appendages, and the activated NF-κB transcription factors can enter the nucleus to promote the expression of NF-κB target genes, such as keratins, cyclin D1, Shh and fox family genes (Schmidt-Ullrich et al., 2006). In different stages of ESG development, these genes are essential (Kunisada et al., 2009). Thus, researchers have sought to determine whether NF-κB could induce the regeneration of ESGs *in vitro*. Zhao et al. (2015) found that human fibroblasts could be directly reprogrammed into SGLCs by introducing NF-κB and Lef-1 (a downstream transcription factor of β-catenin signaling) genes into human fibroblasts. Chen et al. (2019) also noted increased expression of NF-κB during the reprogramming of BM-MSCs into SGLCs by determining the differential expression of miRNAs between BM-MSCs and SGLCs. These results indicate that EDA/EDAR/NF-κB signaling is not only associated with the occurrence and development of ESGs but also plays a vital role in ESG regeneration. However, many other aspects of the EDA/EDAR/NF-κB pathway for ESG regeneration still need to be thoroughly explored, such as receptor activation, ligand binding sites, desensitization, and transportation. It indicates that EDA/EDAR/NF-κB signaling are not only related to the determination and development of ESGs, but also important in ESGs regeneration.

### Shh Signaling Pathway

The Shh signaling pathway plays a vital role in embryonic development and tissue regeneration (Xu et al., 2015). The Shh signaling pathway is downstream of the EDA/EDAR/NF-κB signaling pathway. Some studies have shown that Shh signaling is involved in the development of ESG, especially in the process of ESG induction and/or early development, but not in the process of maturation and/or maintenance (Kunisada et al., 2009; Lu and Fuchs, 2014; Lu et al., 2016). Conversely, many studies have also shown that Shh signaling inactivation does not affect the formation of ESG germ or subsequent ducts, but the secretory coil formation is still blocked in the primary stage (Cui et al., 2014; Cui and Schlessinger, 2015). In the process of ESG cells regeneration, it is unclear whether there is a specific connection between the two experimental results. Liang et al. (2016) reported that Shh is an important factor in conditioned medium that influences the differentiation and the formation of ESG tubule-like structures during the differentiation of amniotic fluid stem cells into SGLCs. However, the underlying mechanism is unknown and the exact role of Shh signaling in ESG morphogenesis remains to be clarified.

### BMP Signaling Pathway

Bone morphogenetic proteins (BMPs) are multi-functional growth factors belonging to the transforming growth factor (TGF)-β superfamily (Botchkarev and Sharov, 2004). Previous experiments have shown that the ESGs in the mouse paws can be converted into HFs by suppressing the BMP signaling (Plikus et al., 2004). Lu et al. (2016) investigated it further and found that the selection of appendages depends on the antagonism between Shh signaling and BMP signaling in different skin areas in the mesenchyme after epidermal bud formation. When the BMP signaling is in the active state, it determines the formation of ESGs. When BMP signaling is weaker than Shh, it determines the formation of HFs. Hu et al. (2019) cocultured EpiSCs with embryonic paw pad tissue, which demonstrated glandular structure. Moreover, BMP4 concentration was detected in the medium and a BMP receptor inhibitor could effectively block the EpiSC differentiation to ESGs (Hu et al., 2019), implying the possibility of BMP4 application in the regeneration of ESGs.

### ERK Signaling Pathway

Epidermal growth factor and FGF, as cytokines, can activate the ERK signaling pathway. EGF can specifically trigger proliferation or differentiation by leading to population-averaged transient or sustained ERK (Marshall, 1995; Santos et al., 2007). By activating ERK through FGFRs, FGF can regulate development, wound healing, and angiogenesis (Ornitz and Itoh, 2015). Some studies have shown that EGF or KGF (also called FGF7) could induce stem cell differentiation into SGLCs (Xu et al., 2016; Kolakshyapati et al., 2017). All of these show that the ERK pathway is important in ESG regeneration.

## Conclusion and Future Perspectives

Recently, skin tissue engineering research has been greatly developed. However, current skin substitutes do not contain skin appendages. Therefore, current skin substitutes can only be used to cover the wound, but cannot play physiological functions of normal skin, which is far from enough for patients with severe burns. Studies on the development, structure and function of ESGs have been intensively conducted. On this basis, ESG regeneration has been studied and great advances have been made. The study of skin tissue engineering is often divided into several aspects of cells, scaffolds and biomolecules, and ESG regeneration research is also similar. In this review, ESG and its regeneration have been systematically reviewed. There are three main categories must be considered in ESG regeneration: stem cells, scaffolds, and possible signaling pathways involved.

It is clear from the works herein reviewed that ESG regeneration research involves combination of different types of stem cells, scaffolds, and signaling pathways. So far, researchers successfully reconstructed SGL structures via a variety of methods. However, whether the 3D-reconstituted ESGs can perform physiological functions needs further verification. In addition, the detailed mechanism of how a variety of biomolecules induces ESG differentiation remains to be further studied. Current methods of regenerating ESGs are inefficient, mainly due to the limited number of stem cells, low cell differentiation efficiency and other unpredictable factors. In conclusion, ESG regeneration research is still at a very early stage. We expect to be able to regenerate ESGs to compensate for the inability of tissue-engineered skin to secrete sweat. With the development of stem cells study, molecular biology and biomaterials, ESG regeneration will be achieved in future.

## Author Contributions

HL conceived and presented the outline of the review. YL collected literature as well as wrote the review. LC, MZ, SX, LD, and XZ revised the manuscript, provided some relevant insights, and made some edits. All authors read and approved the final version of the manuscript.

## Conflict of Interest

The authors declare that the research was conducted in the absence of any commercial or financial relationships that could be construed as a potential conflict of interest.

## Publisher’s Note

All claims expressed in this article are solely those of the authors and do not necessarily represent those of their affiliated organizations, or those of the publisher, the editors and the reviewers. Any product that may be evaluated in this article, or claim that may be made by its manufacturer, is not guaranteed or endorsed by the publisher.

## References

[B1] Amini-Nik S. Dolp R. Eylert G. Datu A. K. Parousis A. Blakeley C. (2018). Stem cells derived from burned skin - The future of burn care. *EBioMed.* 37 509–520. 10.1016/j.ebiom.2018.10.014 30409728 PMC6284415

[B2] Arnaoutova I. George J. Kleinman H. K. Benton G. (2009). The endothelial cell tube formation assay on basement membrane turns 20: state of the science and the art. *Angiogenesis* 12 267–274. 10.1007/s10456-009-9146-4 19399631

[B3] Auerbach R. Lewis R. Shinners B. Kubai L. Akhtar N. (2003). Angiogenesis assays: a critical overview. *Clin. Chem.* 49 32–40. 10.1373/49.1.32 12507958

[B4] Barka T. Gresik E. S. Miyazaki Y. (2005). Differentiation of a mouse submandibular gland-derived cell line (SCA) grown on matrigel. *Exp. Cell. Res.* 308 394–406. 10.1016/j.yexcr.2005.04.025 15922327

[B5] Bissell M. J. Labarge M. A. (2005). Context, tissue plasticity, and cancer: are tumor stem cells also regulated by the microenvironment? *Cancer Cell.* 7 17–23. 10.1016/j.ccr.2004.12.013 15652746 PMC2933216

[B6] Blankenship M. L. (1990). Mite dermatitis other than scabies. *Dermatol. Clin.* 8 265–275. 10.1016/s0733-8635(18)30498-4 2191799

[B7] Boehnke K. Falkowska-Hansen B. Stark H. J. Boukamp P. (2012). Stem cells of the human epidermis and their niche: composition and function in epidermal regeneration and carcinogenesis. *Carcinogenesis* 33 1247–1258. 10.1093/carcin/bgs136 22461521

[B8] Bonnans C. Chou J. Werb Z. (2014). Remodelling the extracellular matrix in development and disease. *Nat. Rev. Mol. Cell Biol.* 15 786–801. 10.1038/nrm3904 25415508 PMC4316204

[B9] Botchkarev V. A. Sharov A. A. (2004). BMP signaling in the control of skin development and hair follicle growth. *Diff. Res. Biol. Divers.* 72 512–526. 10.1111/j.1432-0436.2004.07209005.x 15617562

[B10] Cai S. Pan Y. Han B. Sun T. Z. Sheng Z. Y. Fu X. B. (2011). Transplantation of human bone marrow-derived mesenchymal stem cells transfected with ectodysplasin for regeneration of sweat glands. *Chin. Med. J.* 124 2260–2268. 21933554

[B11] Calvo F. Agudo-Ibáñez L. Crespo P. (2010). The Ras-ERK pathway: understanding site-specific signaling provides hope of new anti-tumor therapies. *Bioessays* 32 412–421. 10.1002/bies.200900155 20414899

[B12] Chen L. Zhang M. Li H. Tang S. Fu X. (2014). Distribution of BrdU label-retaining cells in eccrine sweat glands and comparison of the percentage of BrdU-positive cells in eccrine sweat glands and in epidermis in rats. *Arch. Dermatol. Res.* 306 157–162. 10.1007/s00403-013-1397-7 23907330

[B13] Chen Y. Li Q. Tan Z. Zhang C. Fu X. (2019). MicroRNA-mediated regulation of BM-MSCs differentiation into sweat gland-like cells: targeting NF-kappaB. *J. Mol. Histol.* 50 155–166. 10.1007/s10735-019-09814-2 30783857

[B14] Cluzeau C. Hadj-Rabia S. Jambou M. Mansour S. Guigue P. Masmoudi S. (2011). Only four genes (EDA1, EDAR, EDARADD, and WNT10A) account for 90% of hypohidrotic/anhidrotic ectodermal dysplasia cases. *Hum. Mutat.* 32 70–72. 10.1002/humu.21384 20979233

[B15] Costa A. Naranjo J. D. Londono R. Badylak S. F. (2017). Biologic Scaffolds. *Cold Spring Harb. Perspect. Med.* 7:a025676. 10.1101/cshperspect.a025676 28320826 PMC5580515

[B16] Cotsarelis G. Kaur P. Dhouailly D. Hengge U. Bickenbach J. (1999). Epithelial stem cells in the skin: definition, markers, localization and functions. *Exp. Dermatol.* 8 80–88. 10.1111/j.1600-0625.1999.tb00351.x 10206725

[B17] Cui C. Y. Schlessinger D. (2006). EDA signaling and skin appendage development. *Cell Cycle* 5 2477–2483. 10.4161/cc.5.21.3403 17102627 PMC2860309

[B18] Cui C. Y. Schlessinger D. (2015). Eccrine sweat gland development and sweat secretion. *Exp. Dermatol.* 24 644–650. 10.1111/exd.12773 26014472 PMC5508982

[B19] Cui C. Y. Yin M. Sima J. Childress V. Michel M. Piao Y. (2014). Involvement of Wnt, Eda and Shh at defined stages of sweat gland development. *Development* 141 3752–3760. 10.1242/dev.109231 25249463 PMC4197578

[B20] Diao J. Liu J. Wang S. Chang M. Wang X. Guo B. (2019). Sweat gland organoids contribute to cutaneous wound healing and sweat gland regeneration. *Cell Death Dis.* 10:238. 10.1038/s41419-019-1485-5 30858357 PMC6411741

[B21] Doffinger R. Smahi A. Bessia C. Geissmann F. Feinberg J. Durandy A. (2001). X-linked anhidrotic ectodermal dysplasia with immunodeficiency is caused by impaired NF-kappaB signaling. *Nat. Genet.* 27 277–285. 10.1038/85837 11242109

[B22] Fattahi S. Pilehchian Langroudi M. Akhavan-Niaki H. (2018). Hedgehog signaling pathway: Epigenetic regulation and role in disease and cancer development. *J. Cell. Physiol.* 233 5726–5735. 10.1002/jcp.26506 29380372

[B23] Gaide O. Schneider P. (2003). Permanent correction of an inherited ectodermal dysplasia with recombinant EDA. *Nat. Med.* 9 614–618. 10.1038/nm861 12692542

[B24] Gallo S. Vitacolonna A. Bonzano A. Comoglio P. Crepaldi T. (2019). ERK: a Key Player in the Pathophysiology of Cardiac Hypertrophy. *Int. J. Mol. Sci.* 20:2164. 10.3390/ijms20092164 31052420 PMC6539093

[B25] Gonzalez D. M. Medici D. (2014). Signaling mechanisms of the epithelial-mesenchymal transition. *Sci. Signal.* 7:re8. 10.1126/scisignal.2005189 25249658 PMC4372086

[B26] Hashimoto K. Gross B. G. Lever W. F. (1965). The ultrastructure of the skin of human embryos. I. The intraepidermal eccrine sweat duct. *J. Investig. Dermatol.* 45 139–151. 10.1038/jid.1965.110 5829529

[B27] Hassan W. U. Greiser U. Wang W. (2014). Role of adipose-derived stem cells in wound healing. *Wound Repair Regen.* 22 313–325. 10.1111/wrr.12173 24844331

[B28] Headon D. J. Emmal S. A. Ferguson B. M. Tucker A. S. Justice M. J. Sharpe P. T. (2001). Gene defect in ectodermal dysplasia implicates a death domain adapter in development. *Nature* 414 913–916. 10.1038/414913a 11780064

[B29] Horie N. Yokozeki H. Sato K. (1986). Proteolytic enzymes in human eccrine sweat: a screening study. *Am. J. Physiol.* 250 R691–R698. 10.1152/ajpregu.1986.250.4.R691 3515973

[B30] Hu T. Xu Y. Yao B. Fu X. Huang S. (2019). Developing a Novel and Convenient Model for Investigating Sweat Gland Morphogenesis from Epidermal Stem Cells. *Stem Cells Int.* 2019:4254759. 10.1155/2019/4254759 30863451 PMC6378793

[B31] Huang S. Lu G. Wu Y. Jirigala E. Xu Y. Ma K. (2012). Mesenchymal stem cells delivered in a microsphere-based engineered skin contribute to cutaneous wound healing and sweat gland repair. *J. Dermatol. Sci.* 66 29–36. 10.1016/j.jdermsci.2012.02.002 22398148

[B32] Huang S. Xu Y. Wu C. Sha D. Fu X. (2010). In vitro constitution and in vivo implantation of engineered skin constructs with sweat glands. *Biomaterials* 31 5520–5525. 10.1016/j.biomaterials.2010.03.060 20398932

[B33] Huang S. Yao B. Xie J. Fu X. (2016). 3D bioprinted extracellular matrix mimics facilitate directed differentiation of epithelial progenitors for sweat gland regeneration. *Acta Biomater.* 32 170–177. 10.1016/j.actbio.2015.12.039 26747979

[B34] Huang S. Zhang Y. Tang L. Deng Z. Lu W. Feng F. (2009). Functional bilayered skin substitute constructed by tissue-engineered extracellular matrix and microsphere-incorporated gelatin hydrogel for wound repair. *Tissue Eng. Part A* 15 2617–2624. 10.1089/ten.TEA.2008.0505 19199780

[B35] Kleinman H. K. Martin G. R. (2005). Matrigel: basement membrane matrix with biological activity. *Semin. Cancer Biol.* 15 378–386. 10.1016/j.semcancer.2005.05.004 15975825

[B36] Kolakshyapati P. Li X. Chen C. Zhang M. Tan W. Ma L. (2017). Gene-activated matrix/bone marrow-derived mesenchymal stem cells constructs regenerate sweat glands-like structure in vivo. *Sci. Rep.* 7:17630. 10.1038/s41598-017-17967-x 29247230 PMC5732266

[B37] Kozowski M. Wilczak J. Motyl T. Gajewska M. (2011). Role of extracellular matrix and prolactin in functional differentiation of bovine BME-UV1 mammary epithelial cells. *Pol. J. Vet. Sci.* 14 433–442. 10.2478/v10181-011-0064-1 21957738

[B38] Kumar A. Eby M. T. Sinha S. Jasmin A. Chaudhary P. M. (2001). The ectodermal dysplasia receptor activates the nuclear factor-kappaB, JNK, and cell death pathways and binds to ectodysplasin A. *J. Biol. Chem.* 276 2668–2677. 10.1074/jbc.M008356200 11035039

[B39] Kunisada M. Cui C. Y. Piao Y. Ko M. S. Schlessinger D. (2009). Requirement for Shh and Fox family genes at different stages in sweat gland development. *Hum. Mol. Genet.* 18 1769–1778. 10.1093/hmg/ddp089 19270025 PMC2671986

[B40] Kurata R. Futaki S. Nakano I. Tanemura A. Murota H. Katayama I. (2014). Isolation and characterization of sweat gland myoepithelial cells from human skin. *Cell Struct. Funct.* 39 101–112. 10.1247/csf.14009 25196208

[B41] Lee C. H. Rodeo S. A. Fortier L. A. Lu C. Erisken C. Mao J. J. (2014). Protein-releasing polymeric scaffolds induce fibrochondrocytic differentiation of endogenous cells for knee meniscus regeneration in sheep. *Sci. Transl. Med.* 6:266ra171. 10.1126/scitranslmed.3009696 25504882 PMC4546837

[B42] Leung Y. Kandyba E. Chen Y. B. Ruffins S. Kobielak K. (2013). Label retaining cells (LRCs) with myoepithelial characteristic from the proximal acinar region define stem cells in the sweat gland. *PLoS One* 8:e74174. 10.1371/journal.pone.0074174 24058524 PMC3776797

[B43] Li H. Chen L. Zeng S. Li X. Zhang X. Lin C. (2015a). Matrigel basement membrane matrix induces eccrine sweat gland cells to reconstitute sweat gland-like structures in nude mice. *Exp. Cell Res.* 332 67–77. 10.1016/j.yexcr.2015.01.014 25645942

[B44] Li H. Chen L. Zhang M. Tang S. Fu X. (2013). Three-dimensional culture and identification of human eccrine sweat glands in matrigel basement membrane matrix. *Cell Tissue Res.* 354 897–902. 10.1007/s00441-013-1718-3 23996202

[B45] Li H. Chen L. Zhang M. Xie S. Cheng L. (2018). Expression and localization of Forkhead transcription factor A1 in the three-dimensional reconstructed eccrine sweat glands. *Acta Histochem.* 120 520–524. 10.1016/j.acthis.2018.06.003 29909922

[B46] Li H. Chen L. Zhang M. Zhang B. (2017). Foxa1 gene and protein in developing rat eccrine sweat glands. *J. Mol. Histol.* 48 1–7. 10.1007/s10735-016-9700-5 27787633

[B47] Li H. Fu X. Ouyang Y. Cai C. Wang J. Sun T. (2006). Adult bone-marrow-derived mesenchymal stem cells contribute to wound healing of skin appendages. *Cell Tissue Res.* 326 725–736. 10.1007/s00441-006-0270-9 16906419

[B48] Li H. Li X. Zhang M. Chen L. Zhang B. Tang S. (2015b). Three-dimensional co-culture of BM-MSCs and eccrine sweat gland cells in Matrigel promotes transdifferentiation of BM-MSCs. *J. Mol. Histol.* 46 431–438. 10.1007/s10735-015-9632-5 26189057

[B49] Li H. Zhang M. Chen L. Li X. Zhang B. (2016a). Human eccrine sweat gland cells reconstitute polarized spheroids when subcutaneously implanted with Matrigel in nude mice. *J. Mol. Histol.* 47 485–490. 10.1007/s10735-016-9690-3 27492422

[B50] Li H. Zhang M. Li X. Chen L. Zhang B. Tang S. (2016b). BrdU-label-retaining cells in rat eccrine sweat glands over time. *Acta Histochem.* 118 74–79. 10.1016/j.acthis.2015.11.009 26657518

[B51] Li H. H. Fu X. B. Zhang L. Zhou G. (2008). Comparison of proliferating cells between human adult and fetal eccrine sweat glands. *Arch Dermatol. Res.* 300 173–176. 10.1007/s00403-007-0823-0 18193437

[B52] Liang H. Sun Q. Zhen Y. Li F. Xu Y. Liu Y. (2016). The differentiation of amniotic fluid stem cells into sweat glandlike cells is enhanced by the presence of Sonic hedgehog in the conditioned medium. *Exp. Dermatol.* 25 714–720. 10.1111/exd.13062 27120089

[B53] Liu N. Huang S. Yao B. Xie J. Wu X. Fu X. (2016). 3D bioprinting matrices with controlled pore structure and release function guide in vitro self-organization of sweat gland. *Sci. Rep.* 6:34410. 10.1038/srep34410 27694985 PMC5046070

[B54] Lobitz W. C. Jr. Dobson R. L. (1961). Dermatology: the eccrine sweat glands. *Annu. Rev. Med.* 12 289–298. 10.1146/annurev.me.12.020161.001445 13762949

[B55] Lu C. Fuchs E. (2014). Sweat gland progenitors in development, homeostasis, and wound repair. *Cold Spring Harb. Perspect. Med.* 4:a015222. 10.1101/cshperspect.a015222 24492848 PMC3904096

[B56] Lu C. P. Polak L. Keyes B. E. Fuchs E. (2016). Spatiotemporal antagonism in mesenchymal-epithelial signaling in sweat versus hair fate decision. *Science* 354:aah6102. 10.1126/science.aah6102 28008008 PMC5333576

[B57] Lu C. P. Polak L. Rocha A. S. Pasolli H. A. Chen S. C. Sharma N. (2012). Identification of stem cell populations in sweat glands and ducts reveals roles in homeostasis and wound repair. *Cell* 150 136–150. 10.1016/j.cell.2012.04.045 22770217 PMC3423199

[B58] Ma X. Liu J. Zhu W. Tang M. Lawrence N. Yu C. (2018). 3D bioprinting of functional tissue models for personalized drug screening and in vitro disease modeling. *Adv. Drug Del. Rev.* 132 235–251. 10.1016/j.addr.2018.06.011 29935988 PMC6226327

[B59] Ma Y. Li M. Liu J. Pang C. Zhang J. Li Y. (2018). Location, Isolation, and Identification of Mesenchymal Stem Cells from Adult Human Sweat Glands. *Stem Cells Int.* 2018:2090276. 10.1155/2018/2090276 29983714 PMC6015687

[B60] Maria O. M. Maria O. Liu Y. Komarova S. V. Tran S. D. (2011). Matrigel improves functional properties of human submandibular salivary gland cell line. *Int. J. Biochem. Cell Biol.* 43 622–631. 10.1016/j.biocel.2011.01.001 21216302

[B61] Marshall C. J. (1995). Specificity of receptor tyrosine kinase signaling: transient versus sustained extracellular signal-regulated kinase activation. *Cell* 80 179–185. 10.1016/0092-8674(95)90401-8 7834738

[B62] Mikkola M. L. (2009). Molecular aspects of hypohidrotic ectodermal dysplasia. *Am. J. Med. Genet. A* 149A 2031–2036. 10.1002/ajmg.a.32855 19681132

[B63] Monreal A. W. Ferguson B. M. Headon D. J. Street S. L. Overbeek P. A. Zonana J. (1999). Mutations in the human homologue of mouse dl cause autosomal recessive and dominant hypohidrotic ectodermal dysplasia. *Nat. Genet.* 22 366–369. 10.1038/11937 10431241

[B64] Montagna W. Chase H. B. Lobitz W. C. Jr. (1953). Histology and cytochemistry of human skin. IV. The eccrine sweat glands. *J. Investig. Dermatol.* 20 415–423. 10.1038/jid.1953.52 13069827

[B65] Morimoto Y. Saga K. (1995). Proliferating cells in human eccrine and apocrine sweat glands. *J. Histochem. Cytochem.* 43 1217–1221. 10.1177/43.12.8537637 8537637

[B66] Munger B. L. (1961). The ultrastructure and histophysiology of human eccrine sweat glands. *J. Biophys. Biochem. cytol.* 11 385–402. 10.1083/jcb.11.2.385 14477206 PMC2225150

[B67] Murphy S. V. Atala A. (2014). 3D bioprinting of tissues and organs. *Nat. Biotechnol.* 32 773–785. 10.1038/nbt.2958 25093879

[B68] Mustonen T. Pispa J. Mikkola M. L. Pummila M. Kangas A. T. Pakkasjarvi L. (2003). Stimulation of ectodermal organ development by Ectodysplasin-A1. *Dev. Biol.* 259 123–136. 10.1016/s0012-1606(03)00157-x 12812793

[B69] Niyonsaba F. Suzuki A. Ushio H. Nagaoka I. Ogawa H. Okumura K. (2009). The human antimicrobial peptide dermcidin activates normal human keratinocytes. *Br. J. Dermatol.* 160 243–249. 10.1111/j.1365-2133.2008.08925.x 19014393

[B70] Nolte S. V. Xu W. Rennekampff H. O. Rodemann H. P. (2008). Diversity of fibroblasts–a review on implications for skin tissue engineering. *Cells Tissues Organs.* 187 165–176. 10.1159/000111805 18042973

[B71] Nusse R. Clevers H. (2017). Wnt/β-Catenin Signaling, Disease, and Emerging Therapeutic Modalities. *Cell* 169 985–999. 10.1016/j.cell.2017.05.016 28575679

[B72] Oeckinghaus A. Hayden M. S. Ghosh S. (2011). Crosstalk in NF-κB signaling pathways. *Nat. Immunol.* 12 695–708. 10.1038/ni.2065 21772278

[B73] Okada T. Konishi H. Ito M. Nagura H. Asai J. (1988). Identification of secretory immunoglobulin A in human sweat and sweat glands. *J. Investig. Dermatol.* 90 648–651. 10.1111/1523-1747.ep12560807 3283249

[B74] Ornitz D. M. Itoh N. (2015). The Fibroblast Growth Factor signaling pathway. *Wiley Interdiscip. Rev. Dev. Biol.* 4 215–266. 10.1002/wdev.176 25772309 PMC4393358

[B75] Pati F. Jang J. Ha D. H. Won Kim S. Rhie J. W. Shim J. H. (2014). Printing three-dimensional tissue analogues with decellularized extracellular matrix bioink. *Nature communications.* 5 3935. 10.1038/ncomms4935 24887553 PMC4059935

[B76] Petrakova O. S. Ashapkin V. V. Voroteliak E. A. Bragin E. Y. Shtratnikova V. Y. Chernioglo E. S. (2012). Effect of 3D Cultivation Conditions on the Differentiation of Endodermal Cells. *Acta Nat.* 4 47–57. 10.32607/20758251-2012-4-4-47-57 PMC3548173 23346379

[B77] Plikus M. Wang W. P. Liu J. Wang X. Jiang T. X. Chuong C. M. (2004). Morpho-regulation of ectodermal organs: integument pathology and phenotypic variations in K14-Noggin engineered mice through modulation of bone morphogenic protein pathway. *Am. J. Pathol.* 164 1099–1114. 10.1016/s0002-9440(10)63197-5 14982863 PMC1614723

[B78] Pontiggia L. Biedermann T. Bottcher-Haberzeth S. Oliveira C. Braziulis E. Klar A. S. (2014). De novo epidermal regeneration using human eccrine sweat gland cells: higher competence of secretory over absorptive cells. *J. Investig. Dermatol.* 134 1735–1742. 10.1038/jid.2014.30 24448031

[B79] Rittie L. Sachs D. L. Orringer J. S. Voorhees J. J. Fisher G. J. (2013). Eccrine sweat glands are major contributors to reepithelialization of human wounds. *Am. J. Pathol.* 182 163–171. 10.1016/j.ajpath.2012.09.01 23159944 PMC3538027

[B80] Routledge D. Scholpp S. (2019). Mechanisms of intercellular Wnt transport. *Development* 146:dev176073. 10.1242/dev.176073 31092504

[B81] Saga K. (2002). Structure and function of human sweat glands studied with histochemistry and cytochemistry. *Prog. Histochem. Cytochem.* 37 323–386. 10.1016/s0079-6336(02)80005-5 12365351

[B82] Santos S. D. Verveer P. J. Bastiaens P. I. (2007). Growth factor-induced MAPK network topology shapes Erk response determining PC-12 cell fate. *Nat. Cell Biol.* 9 324–330. 10.1038/ncb1543 17310240

[B83] Sato K. (1977). Pharmacology and function of the myoepithelial cell in the eccrine sweat gland. *Experientia* 33 631–633. 10.1007/bf01946542 405245

[B84] Sato K. Kang W. H. Saga K. Sato K. T. (1989). Biology of sweat glands and their disorders. I. Normal sweat gland function. *J. Am. Acad. Dermatol.* 20 537–563. 10.1016/s0190-9622(89)70063-3 2654204

[B85] Sato K. Sato F. (1994). Interleukin-1 alpha in human sweat is functionally active and derived from the eccrine sweat gland. *Am. J. Physiol.* 266 R950–R959. 10.1152/ajpregu.1994.266.3.R950 8160891

[B86] Schittek B. Hipfel R. Sauer B. Bauer J. Kalbacher H. Stevanovic S. (2001). Dermcidin: a novel human antibiotic peptide secreted by sweat glands. *Nat. Immunol.* 2 1133–1137. 10.1038/ni732 11694882

[B87] Schmidt-Ullrich R. Aebischer T. Hülsken J. Birchmeier W. Klemm U. Scheidereit C. (2001). Requirement of NF-kappaB/Rel for the development of hair follicles and other epidermal appendices. *Development* 128 3843–3853. 10.1242/dev.128.19.3843 11585809

[B88] Schmidt-Ullrich R. Tobin D. J. Lenhard D. Schneider P. Paus R. Scheidereit C. (2006). NF-kappaB transmits Eda A1/EdaR signalling to activate Shh and cyclin D1 expression, and controls post-initiation hair placode down growth. *Development* 133 1045–1057. 10.1242/dev.02278 16481354

[B89] Sheng Z. Fu X. Cai S. Lei Y. Sun T. Bai X. (2009). Regeneration of functional sweat gland-like structures by transplanted differentiated bone marrow mesenchymal stem cells. *Wound Repair Regen.* 17 427–435. 10.1111/j.1524-475X.2009.00474.x 19660052

[B90] Shevchenko R. V. Eeman M. Rowshanravan B. Allan I. U. Savina I. N. Illsley M. (2014). The in vitro characterization of a gelatin scaffold, prepared by cryogelation and assessed in vivo as a dermal replacement in wound repair. *Acta Biomater.* 10 3156–3166. 10.1016/j.actbio.2014.03.027 24704695

[B91] Shibasaki M. Wilson T. E. Crandall C. G. (2006). Neural control and mechanisms of eccrine sweating during heat stress and exercise. *J. Appl. Physiol.* 100 1692–1701. 10.1152/japplphysiol.01124.2005 16614366

[B92] Shikiji T. Minami M. Inoue T. Hirose K. Oura H. Arase S. (2003). Keratinocytes can differentiate into eccrine sweat ducts in vitro: involvement of epidermal growth factor and fetal bovine serum. *J. Dermatol. Sci.* 33 141–150. 10.1016/j.jdermsci.2003.09.004 14643519

[B93] Sisto M. Barca A. Lofrumento D. D. Lisi S. (2016). Downstream activation of NF-κB in the EDA-A1/EDAR signalling in Sjögren’s syndrome and its regulation by the ubiquitin-editing enzyme A20. *Clin. Exp. Immunol.* 184 183–196. 10.1111/cei.12764 26724675 PMC4837237

[B94] Srivastava A. K. Durmowicz M. C. Hartung A. J. Hudson J. Ouzts L. V. Donovan D. M. (2001). Ectodysplasin-A1 is sufficient to rescue both hair growth and sweat glands in Tabby mice. *Hum. Mol. Genet.* 10 2973–2981. 10.1093/hmg/10.26.2973 11751679

[B95] Srivastava A. K. Pispa J. Hartung A. J. Du Y. Ezer S. Jenks T. (1997). The Tabby phenotype is caused by mutation in a mouse homologue of the EDA gene that reveals novel mouse and human exons and encodes a protein (ectodysplasin-A) with collagenous domains. *Proc. Natl. Acad. Sci. U. S. A.* 94 13069–13074. 10.1073/pnas.94.24.13069 9371801 PMC24264

[B96] Steinhart Z. Angers S. (2018). Wnt signaling in development and tissue homeostasis. *Development* 145:dev146589. 10.1242/dev.146589 29884654

[B97] Sun S. Xiao J. Huo J. Geng Z. Ma K. Sun X. (2018). Targeting ectodysplasin promotor by CRISPR/dCas9-effector effectively induces the reprogramming of human bone marrow-derived mesenchymal stem cells into sweat gland-like cells. *Stem Cell Res. Ther.* 9:8. 10.1186/s13287-017-0758-0 29329593 PMC5766979

[B98] Tao R. Sun T. J. Han Y. Q. Xu G. Liu J. Han Y. F. (2014). Epimorphin-induced differentiation of human umbilical cord mesenchymal stem cells into sweat gland cells. *Eur. Rev. Med. Pharmacol. Sci.* 18 1404–1410. 24867521

[B99] Theocharis A. D. Skandalis S. S. Gialeli C. Karamanos N. K. (2016). Extracellular matrix structure. *Adv. Drug Deliv. Rev.* 97 4–27. 10.1016/j.addr.2015.11.001 26562801

[B100] Vierbuchen T. Ostermeier A. Pang Z. P. Kokubu Y. Sudhof T. C. Wernig M. (2010). Direct conversion of fibroblasts to functional neurons by defined factors. *Nature* 463 1035–1041. 10.1038/nature08797 20107439 PMC2829121

[B101] Wang R. Wang Y. Yao B. Hu T. Li Z. Liu Y. (2019). Redirecting differentiation of mammary progenitor cells by 3D bioprinted sweat gland microenvironment. *Burns Trauma* 7:29. 10.1186/s41038-019-0167-y 31559316 PMC6755689

[B102] Wang Y. Liu Z. Y. Zhao Q. Sun T. Z. Ma K. Fu X. B. (2013). Future application of hair follicle stem cells: capable in differentiation into sweat gland cells. *Chin. Med. J.* 126 3545–3552. 24034106

[B103] Watt F. M. Huck W. T. (2013). Role of the extracellular matrix in regulating stem cell fate. *Nat. Rev. Mol. Cell Biol.* 14 467–473. 10.1038/nrm3620 23839578

[B104] Xin M. Ji X. De La Cruz L. K. Thareja S. Wang B. (2018). Strategies to target the Hedgehog signaling pathway for cancer therapy. *Med. Res. Rev.* 38 870–913. 10.1002/med.21482 29315702

[B105] Xu M. Gong A. Yang H. George S. K. Jiao Z. Huang H. (2015). Sonic hedgehog-glioma associated oncogene homolog 1 signaling enhances drug resistance in CD44(+)/Musashi-1(+) gastric cancer stem cells. *Cancer Lett.* 369 124–133. 10.1016/j.canlet.2015.08.005 26276718

[B106] Xu M. Horrell J. Snitow M. Cui J. Gochnauer H. Syrett C. M. (2017). WNT10A mutation causes ectodermal dysplasia by impairing progenitor cell proliferation and KLF4-mediated differentiation. *Nat. Commun.* 8:15397. 10.1038/ncomms15397 28589954 PMC5467248

[B107] Xu Y. Hong Y. Xu M. Ma K. Fu X. Zhang M. (2016). Role of Keratinocyte Growth Factor in the Differentiation of Sweat Gland-Like Cells From Human Umbilical Cord-Derived Mesenchymal Stem Cells. *Stem Cells Transl. Med.* 5 106–116. 10.5966/sctm.2015-0081 26574554 PMC4704873

[B108] Xu Y. Huang S. Ma K. Fu X. Han W. Sheng Z. (2012). Promising new potential for mesenchymal stem cells derived from human umbilical cord Wharton’s jelly: sweat gland cell-like differentiative capacity. *J. Tissue Eng. Regen. Med.* 6 645–654. 10.1002/term.468 21916019

[B109] Yanagawa S. Yokozeki H. Sato K. (1986). Origin of periodic acid-Schiff-reactive glycoprotein in human eccrine sweat. *J. Appl. Physiol.* 60 1615–1622. 10.1152/jappl.1986.60.5.1615 3710980

[B110] Yao B. Song W. Li Z. Hu T. Wang R. Wang Y. (2018). Irf6 directs glandular lineage differentiation of epidermal progenitors and promotes limited sweat gland regeneration in a mouse burn model. *Stem Cell Res. Ther.* 9:179. 10.1186/s13287-018-0929-7 29973266 PMC6033224

[B111] Yao B. Wang R. Wang Y. Zhang Y. Hu T. Song W. (2020). Biochemical and structural cues of 3D-printed matrix synergistically direct MSC differentiation for functional sweat gland regeneration. *Sci. Adv.* 6:eaaz1094. 10.1126/sciadv.aaz1094 32181358 PMC7056319

[B112] Yao B. Xie J. Liu N. Hu T. Song W. Huang S. (2019). Direct reprogramming of epidermal cells toward sweat gland-like cells by defined factors. *Cell Death Dis.* 10:272. 10.1038/s41419-019-1503-7 30894517 PMC6426881

[B113] Zhang C. Chen Y. Fu X. (2015). Sweat gland regeneration after burn injury: is stem cell therapy a new hope? *Cytotherapy* 17 526–535. 10.1016/j.jcyt.2014.10.016 25533933

[B114] Zhang M. Li H. Chen L. Fang S. Xie S. Lin C. (2018). Three-dimensional reconstructed eccrine sweat glands with vascularization and cholinergic and adrenergic innervation. *J. Mol. Histol.* 49 339–345. 10.1007/s10735-018-9773-4 29667149

[B115] Zhao Z. Xu M. Wu M. Ma K. Sun M. Tian X. (2015). Direct reprogramming of human fibroblasts into sweat gland-like cells. *Cell Cycle* 14 3498–3505. 10.1080/15384101.2015.1093707 26566868 PMC4825576

